# Freeze-Dried Lopinavir-Loaded Nanostructured Lipid Carriers for Enhanced Cellular Uptake and Bioavailability: Statistical Optimization, in Vitro and in Vivo Evaluations

**DOI:** 10.3390/pharmaceutics11020097

**Published:** 2019-02-25

**Authors:** Arshad Ali Khan, Jahanzeb Mudassir, Safia Akhtar, Vikneswaran Murugaiyah, Yusrida Darwis

**Affiliations:** 1 School of Pharmaceutical Sciences, Universiti Sains Malaysia, 11800 Penang, Malaysia; arshad_pharm@yahoo.co.in (A.A.K.); jahanzebmudassir@hotmail.com (J.M.); safia.pharm@gmail.com (S.A.); vicky@usm.my (V.M.); 2 Faculty of Engineering Technology, Universiti Malaysia Pahang, 26300 Kuantan, Pahang, Malaysia; 3 Department of Pharmaceutics, Faculty of Pharmacy, Bahauddin Zakariya University, Multan 66000, Pakistan; 4 Department of Endocrinology and Metabolism, University of Virginia, Charlottesville, VA 22903, USA

**Keywords:** lopinavir, lipid-based formulations, cellular uptake, factorial design

## Abstract

Nanostructured lipid carriers (NLCs) loaded with lopinavir (LPV) were prepared by the high-shear homogenization method. The LPV-NLCs formulations were freeze-dried using trehalose as a cryoprotectant. In vitro release studies in simulated gastric fluid (pH 1.2) and simulated intestinal fluid (pH 6.8) showed a burst release. The optimized freeze-dried formulation (LPV-NLC-7-Tres) had a particle size (PS), polydispersity index (PdI), zeta potential (ZP) and % entrapment efficiency (%EE) of 286.8 ± 1.3 nm, 0.413 ± 0.017, −48.6 ± 0.89 mV and 88.31 ± 2.04%, respectively. The optimized formulation observed by transmission and scanning electron microscopes showed a spherical shape. Differential scanning calorimetry study revealed the absence of chemical interaction between the drug and lipids. In vitro cellular uptake study using Caco-2 cell line showed a higher LPV uptake from LPV-NLC-7-Tres formulation compared to the free LPV-suspension. The 6-month stability study showed a minimum rise of ~40 nm in PS, while no significant changes in PdI, ZP and drug content of the LPV-NLC-7-Tres formulation stored at 5 °C ± 3 °C. The bioavailability of LPV following oral administration of LPV-NLC-7-Tres in male Wistar rats was found 6.98-fold higher than the LPV-suspension. In conclusion, the nanostructure lipid carriers are potential carriers for improving the oral bioavailability of lopinavir.

## 1. Introduction

Acquired immune deficiency syndrome (AIDS) is a variety of complications caused by the human immunodeficiency virus (HIV) infection. HIV is mostly localized and replicated in the cell (monocytes and CD4+ T lymphocytes), and anatomical (lymphatic system, central nervous system and genitals) levels. Currently, the majority of the available anti-HIV drugs are suppressing the viral replication within the peripheral blood circulation [1]. Therefore, targeting the antiretroviral drug to a system that serves as a viral reservoir will have some advantages with a reasonable approach.

Lopinavir (LPV) is an antiretroviral drug of the protease inhibitor class. It is prescribed as an effective treatment for HIV-1 infections in a combination therapy [1]. LPV exhibits limited oral bioavailability due to poor aqueous solubility (0.01 mg/mL), high P-glycoprotein efflux and extensive first-pass metabolism by liver microsomal enzyme cytochrome P450 (CYP3A4) [2]. Consequently, LPV fails to attain sufficient therapeutic concentration in the systemic circulation, when administered alone. In order to improve the oral bioavailability, LPV is currently marketed as a fixed dose coformulation with ritonavir, under the names of Aluvia^®^ and Kaletra^®^ [3]. Ritonavir is an analogue of LPV that enhances the oral bioavailability of LPV due to its inhibitory effect on CYP3A4 and P-gp [3,4]. However, the use of ritonavir in the combination therapy may cause gastrointestinal intolerance, glucose intolerance, perioral paresthesia and lipid elevation [4]. Hence, it is inevitable to develop LPV formulation without coadministration of ritonavir to improve the oral bioavailability of LPV.

In recent years, lipid-based nanoformulations such as nanostructured lipid carriers (NLCs) have shown great potential in delivering the therapeutic agents to the intestinal lymphatic system and to avoid the first pass metabolism [5,6]. NLCs are employed as an alternative to solid lipid nanoparticles (SLNs) due to its controlled release properties and greater chemical and physical stability. It minimizes the limitations of SLNs, including lower drug loading and formulation instability due to polymorphic modification of similar lipid molecules [7]. Based on Biopharmaceutics Classification System, LPV is a class II drug, which restricts its absorption via intestinal membrane. NLCs may overcome the solubility related absorption formulation by enhancing the saturation solubility of loaded LPV and its dissolution. In addition, NLCs possess more space for drug payload, which may help in reducing the drug dose. Besides that, NLCs also stimulates the formation of chylomicron in enterocytes which leads to the absorption of NLCs by the intestinal lymphatic [8]. The instruments and processing steps such as high shear homogenizer and lyophilizer used in the preparation of stable NLCs formulations can be easily employed in the pharmaceutical industry for the large-scale production.

In the present study, LPV-NLCs formulation for oral delivery was developed using Compritol 888 ATO^®^ (i.e., glyceryl behenate) as the solid lipid, and oleic acid as the liquid lipid. LPV-NLC formulations were prepared using hot high shear homogenization methods and optimized statistically using 2^4^ full factorial design. The formulations were characterized in term of particle size (PS), polydispersity index (PdI), zeta potential (ZP), percent of entrapment efficiency (%EE) and percent yield. The selected LPV-NLCs formulations were freeze-dried using cryoprotectant to improve the stability. In vitro release studies of the selected freeze dried LPV-NLC-Tres formulations were carried out in alkaline and acidic media. The in vitro cellular uptake of LPV from the optimized LPV-NLC-Tres formulation was performed using Caco-2 cell lines. The in vivo study was carried out using male Wistar rats to evaluate the oral bioavailability of LPV.

## 2. Materials and Methods

### 2.1. Chemicals

Lopinavir was obtained from Ranbaxy (Mohali, India). Compritol 888 ATO^®^ (glycerol dibehenate EP- glyceryl behenate NF), was procured from Gattefosse (Saint-Priest, France). Sephadex^®^ G-25, trehalose and fetal bovine serum (FBS) were procured from Sigma-Aldrich (St. Louis, MO, USA). Oleic acid was obtained from R&M Chemicals (Essex, UK). Poloxamer 188 (Pluronic^®^ F-68) was purchased from Molekula (Dorset, UK). Polysorbate 80 (Tween^®^ 80) was purchased from Euro chemo-pharma Sdn. Bhd. (Penang, Malaysia). The Caco-2 cell line was obtained from ATCC (Manassas, VA, USA). The Dulbecco’s modified eagle medium (DMEM) and trypsin 0.25% were purchased from GE healthcare life sciences (Logan, UT, USA). Penicillin-streptomycin solution 100× was obtained from Biowest (Nuaillé, France). Passive lysis buffer (5×) was purchased from Promega (Madison, WI, USA).

### 2.2. Preparation of NLCs and LPV-NLCs

NLCs were prepared using the hot high shear homogenization method [9]. The lipid phase consisted of Compritol 888 ATO^®^ and oleic acid was melted at 85 °C. The aqueous phase consisted of surfactants (Poloxamer 188 and Tween^®^ 80) at a ratio of 1:1 in double-distilled water was also heated to 85 °C. The lipid and aqueous phases were mixed and homogenized at 24,000 rpm for 20 or 30 min using IKA^®^, T 25 digital Ultra-Turrax^®^ homogenizer (Staufen, Germany). The preparation was then left to cool at room temperature (25 °C ± 2 °C) before further characterization.

LPV-NLCs formulations were prepared by adding LPV to the lipid phase. Then the above procedure was followed to produce LPV-NLCs formulations.

### 2.3. Optimization of Various Variables using 2^4^ Full Factorial Design

During the preparation of NLCs, the effect of independent variables on the dependent variables (Table 1) was evaluated and optimized using a 2^4^ full factorial design to find the best combination to produce the NLCs. The effects of four independent variables (at two levels) such as the homogenization time (A), concentrations of solid-lipid (B), liquid-lipid (C) and surfactant concentrations (D) on the dependent variables (i.e., PS, PdI, and ZP) were investigated. Preliminary experiments were carried out to select the true low and high levels of independent variables.

The data obtained were analyzed using the Design Expert software to quantify the effects of independent variables. The significant effect of each independent variable and their interactions on dependent variables was evaluated using ANOVA. The experimental data obtained from the dependent variable response were the results of the effect of independent variables. The data generated from the two-level experimental design was sufficient to fit the following polynomial Equation (1):

X = β_0_ + β_1_A + β_2_B + β_3_C + β_4_D + β_12_AB + β_13_AC + β_14_ AD + β_23_BC + β_24_BD + β_34_CD (1)

where X is a dependent variable, β_0_ is an intercept term and β_1_–β_34_ are coefficients for the independent factors A, B, C, D and their interactions. 3D response surface plots were generated using factorial 2^4^ modules of design-expert^®^ software.

### 2.4. Selections of NLCs using the Desirability Function

The desirability function was used for the selection of the NLCs. Accordingly, the obtained results of responses such as PS, PdI and ZP were fitted into the desirability model of design-expert^®^ software (Version 11, Stat-Ease, Inc., Minneapolis, MN, USA). The desirability value 1 signifies as an acceptable value (most desired value) for the responses, while the desirability value 0 signifies as an unacceptable value

### 2.5. Characterization of Formulations

#### Nanoparticulate Properties

The PS and PdI of formulations were measured using Photon Correlation Spectroscopy (PCS) (Zetasizer 1000HS/3000HS, Malvern Instrument, Malvern, UK). Samples were diluted with filtered, purified water to weaken the PS and PdI opalescence

The ZP was determined using Zetasizer Nano series, Nano Z (red badge) (Malvern Instrument, Malvern, UK). Samples were also diluted with filtered, purified water as above.

### 2.6. Drug Content Estimation

Free LPV was separated from LPV-NLCs formulations using Sephadex G25 columns. LPV-NLCs dispersion (1 mL) was placed on the top of the Sephadex G25 column and centrifuged for 2 min at 2000 rpm. The obtained eluent containing LPV-NLCs were freeze-dried using Labconco, FreeZone Freeze Dry Systems (Kansas City, MO, USA) at −40 °C for 24 h. The amount of LPV encapsulated in the freeze-dried LPV-NLCs sample was analyzed by HPLC.

The HPLC analysis was performed using a Shimadzu chromatography system with LC 20AD delivery pump (Kyoto, Japan), and a Phenomenex C18 column (250 × 4.60 mm). The mobile phase consisted of acetonitrile/0.02 M ammonium acetate buffer at a ratio of 65:35 (*v*/*v*) with a flow rate of 1 mL/min and a column temperature of 40 °C. The injection volume was 20µL and the absorption wavelength was set at 210 nm.

The % yield was determined by freeze-drying the required amount of LPV-NLC sample and the weight of freeze-dried LPV-NLCs sample was measured

The percentage entrapment efficiency (% EE) and % yield were calculated using the following equations:

(2) $$\%\mathrm{EE}=\frac{\mathrm{Weight}\;\mathrm{of}\;\mathrm{encapsulated}\;\mathrm{LPV}}{\mathrm{Initial}\;\mathrm{weight}\;\mathrm{of}\;\mathrm{LPV}}\times 100$$

(3) $$\%\mathrm{Yield}=\frac{\mathrm{Weight}\;\mathrm{of}\;\mathrm{NLCs}\;\mathrm{recovered}}{\mathrm{Initial}\;\mathrm{weight}\;\mathrm{of}\;\mathrm{LPV}\;\mathrm{and}\;\mathrm{excipients}}\times 100.$$

### 2.7. Freeze-Drying Study

#### 2.7.1. Screening of Cryoprotectants

Several cryoprotectants such as mannitol, sorbitol, sucrose and trehalose were screened to find the best cryoprotectant to protect the LPV-NLC during freeze drying. Different ratios of total lipids (solid and liquid): cryoprotectant at 1:2, 1:4, 1:6 and 1:8 were studied. The mixture was freeze-dried using Labconco, FreeZone Freeze Dry Systems (Kansas City, MO, USA) at −40 °C for 24 h. The lyophilized samples were reconstituted in distilled water and analyzed in term of mean PS and PdI. The cryoprotectant, that produced the smallest PS and PdI was selected for further study.

#### 2.7.2. Method of Cryoprotectant Addition

Two different methods of cryoprotectant addition into the LPV-NLCs formulation were investigated for the screening of the best lipid and cryoprotectant ratios. In the first method, the selected cryoprotectant was added after homogenization. The LPV-NLCs formulation (2 mL) was mixed with the cryoprotectant at lipids: cryoprotectant ratios of 1:2, 1:4, 1:6 and 1:8 *w*/*w*. The LPV-NLC formulations were freeze-dried using Labconco, FreeZone Freeze Dry Systems (Kansas City, MO, USA) at −40 °C for 24 h. In the second method, the selected cryoprotectant was added during the homogenization process. The required amount of cryoprotectant was dissolved in the aqueous phase. The mixture was added to the lipid phase and then homogenized to produce LPV-NLCs formulation. The formulation was freeze-dried and characterized.

### 2.8. In Vitro Release Study

In vitro release studies of LPV from the selected freeze-dried formulations (LPV-NLC-Tres) were performed under sink condition for 12 h in simulated gastric fluid (SGF) (pH 1.2) and simulated intestinal fluid (SIF) (pH 6.8) both without the enzyme, but containing 1% poloxamer 188 [10,11]. The LPV-NLC-Tres containing 3 mg of LPV were placed in the 150 mL dissolution media and magnetically stirred at a rate of 100rpm and temperature of 37.5 ± 0.5 °C. Samples were withdrawn at predetermined time intervals. After each sampling, the same volume of fresh dissolution media was added to maintain the sink condition. For comparison, a similar procedure of in vitro release study was also conducted for LPV suspension containing 0.5% *w*/*v* methylcellulose as a suspending agent. The LPV concentrations in the samples were determined using HPLC. The LPV-NLC-Tres formulation, which showed the highest release of LPV was chosen for further study

### 2.9. Differential Scanning Calorimetry (DSC)

DSC was performed using a Perkin-Elmer Pyris 6 DSC (Beaconsfield, UK). The required amount of samples was weighed in an aluminum pan and then sealed using crimper press (Perkin-Elmer; Beaconsfield, UK). The samples were scanned at the rate of 10 °C/min from 0 to 150 °C by purging helium at the rate of 20 mL/min. All samples were scanned in triplicate.

### 2.10. Powder X-ray Diffraction Analysis (PXRD)

The PXRD θ/2θ analysis was performed using powder X-ray diffractometer (High-resolution X-ray diffractometer system; model: PANalytical X’Pert PRO MRD PW3040; Almelo, Netherlands) applying Cu Kα radiation. The samples were subjected to run at a heating rate of 5 °C/min, over a temperature range of 2–60 °C.

### 2.11. Investigations using an Electron Microscope

A sample of LPV-NLCs in a form of liquid (before freeze drying) was smeared on a copper grid (400 mesh) followed by 2% phosphotungstic acid negative staining, then air dried. The morphology of the sample was visualized by transmission electron microscopy (TEM) (Philips CM12; Eindhoven, Netherlands).

The freeze-dried LPV-NLCs without trehalose and optimized LPV-NLC-Tres samples were separately mounted on an aluminum stub and coated with gold in a sputtering device at 15 mA for 15 min. The morphology of the sample was examined using scanning electron microscopy (SEM) (Leo Supra 50 VP field Emission SEM, Carl-Zeiss SMT; Oberkochen, Germany).

### 2.12. Cellular Uptake Study

The Caco-2 cells of passages between 20 and 25 were used in this experiment. Three different dilutions of the optimized LPV-NLC-Tres were prepared in DMEM media (without antibiotic and serum), which comprised of 8.52, 12.79 and 25.58 μg of LPV per 200 μL. Similar dilutions were prepared for the LPV suspension as a control. The Caco-2 cells were seeded in 48-well plates, at a density of 60,000 cells per well with DMEM complete media until the growth of cells becomes 85% or more confluent and formed the monolayer. Subsequently, the DMEM complete media inside the wells was replaced with the prepared dilutions of optimized LPV-NLC-Tres and LPV-suspension, then incubated at 37 °C for 6 h. After the incubation, the test samples were pipetted out from each well and Caco-2 monolayer was washed with phosphate-buffered saline (PBS) for three times to remove residual test samples and dead cells. The absorbed LPV in the Caco-2 cells was extracted by adding 0.2 mL of passive lysis buffer and followed by 0.2 mL methanol into each well. The mixtures were transferred into Eppendorf tubes, then centrifuged at 12,000 rpm for 10 min to separate the lysed Caco-2 cells. The supernatants were collected and LPV concentration was determined by HPLC.

### 2.13. Oral Bioavailability Study

The oral bioavailability study was carried out using male Wister rats weighing 250 ± 20 g. The rats were kept under controlled laboratory conditions at 25 ± 2 °C and 60 ± 5% RH. The rats were retained in the polypropylene cages, with free access to standard laboratory diet and drinking water. The rats were fasted overnight before the experiment. The entire procedures of the experiment were approved by the Animal Ethics Committee Universiti Sains Malaysia, Penang, Malaysia (USM/Animal Ethic Approval/2014/(604). Two groups of animals with each group containing 6 rats were used for the study [11]. The first group was given the optimized LPV-NLC-Tres formulation and the second group was given the LPV-suspension. The optimized LPV-NLC-Tres formulation and LPV-suspension were administered orally at a dose of 12 mg/kg of LPV. Blood samples (0.3 mL) were withdrawn from the tail vein at 0, 0.25, 0.5, 1, 2, 4, 6, 8, 12 and 24 h, post oral administration. The samples were transferred into heparinized Eppendorf tubes, then centrifuge at 5000 rpm for 15 min. The separated plasma was stored in a deep freezer (−80 °C) for further analysis by HPLC method to determine the LPV concentration in the rat plasma. The LPV detection was performed at 210nm, using a Phenomenex C18 (250 × 4.60 mm, 5 µ) column. Acetonitrile/0.02 M ammonium acetate buffer in a ratio of 65:35 (*v/v*) was used as a mobile phase at a flow rate of 1 mL/min and a column temperature of 40 °C. The plasma sample (0.1 mL) was mixed with 500 µL of acetonitrile in an Eppendorf tube followed by addition of 50 µL mefenamic acid as an internal standard. The obtained mixture was vortexed for 2 min and centrifuged for 15 min at 5000 rpm to separate the organic phase from the plasma sample residue. The organic phase was collected in a V-vial and then evaporated under nitrogen gas. The dried residue was dissolved in 100 µL acetonitrile and vortexed for 2 min followed by centrifugation for 5 min at 12000 rpm. The supernatant was collected and 20 µL was injected into the HPLC system to analyze the LPV concentration in the plasma sample.

### 2.14. Stability Studies

The stability study of the optimized freshly prepared freeze-dried LPV-NLC-Tres formulation was carried out according to the ICH guidelines Q1A(R2). The samples were packed in tightly closed vials and stored at the three different conditions 5 ± 3 °C (refrigerator), 25 ± 2 °C/60 ± 5% RH (room condition), and 40 ± 2 °C/75 ± 5% RH (stability chamber). The stability of the optimized LPV-NLC-Tres formulation in term PS, PdI, ZP and drug content were examined at 0, 1, 3 and 6 months.

### 2.15. Statistical Analysis

Statistical data analysis was performed using SPSS^®^ statistical software (Version 22; Armonk, NY, USA). The pharmacokinetic parameters of formulations studied were determined using PK solutions 2.0^®^ noncompartmental pharmacokinetic data analysis software (Windows 2.0.6, Excel). All values were expressed as the mean and standard deviation (mean ± SD) and the differences were considered statistically significant when *p* < 0.05.

## 3. Results and Discussion

### 3.1. The 2^4^ Full Factorial Design

The independent variables such as homogenization time (A), solid lipid (B), liquid lipid (C) and surfactant concentration (D) were optimized using 2^4^ full factorial design. The effect of independent variables on the dependent variables i.e., PS, PdI and ZP of NLCs are shown in Table 2. Equations (4)–(6) show the significant effect of each independent variable and their interaction on dependent variables. The negative or positive sign before each independent factor and their interaction in the equations denote negative or positive impact toward the response, respectively.

PS = 123.87 − 18.95A − 10.30 B − 5.10C + 28.59D + 7.24 AB + 8.45 AC − 8.57 AD + 17.49BC − 15.96 BD − 5.89CD(4)

PdI = 0.46 + 0.020B + 0.021C + 0.020AC + 0.023BC + 0.011BD + 0.022CD(5)

ZP = −43.84 − 0.79A + 1.06B − 4.04D − 1.59AD − 2.01DC + 3.60BD(6)

#### 3.1.1. Effects of Variables on the Mean Particle Size

**Figure 1:**
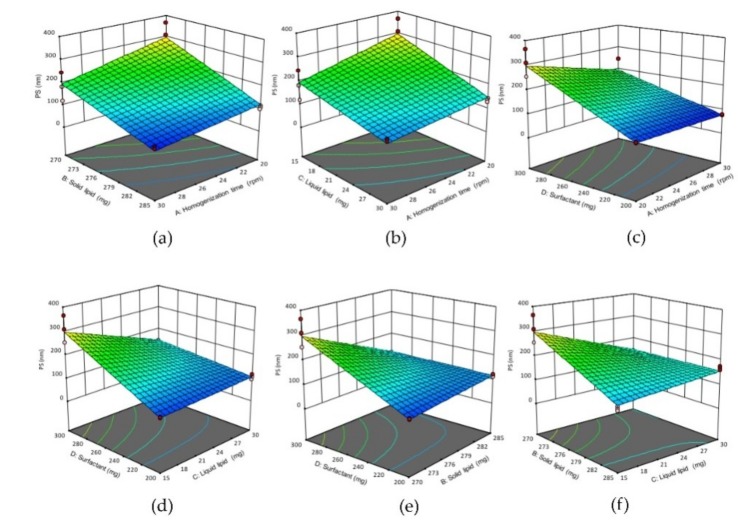
3D response surface plots showing the effect of interactions (**a**) AB, (**b**) AC, (**c**) AD, (**d**) CD, (**e**) BD and (**f**) BC on the PS of NLCs. (**A**) Homogenization time, (**B**) solid lipid concentration, (**C**) liquid lipid concentration, (**D**) surfactant concentration.

The PS of formulations was in the range of 77.68 ± 2.29 nm to 309.86 ± 56.24 nm (Table 2). Equation (4) shows factors A (homogenization time), B (solid lipid concentration) and C (liquid lipid concentration) have a negative impact on PS of NLCs. Thus, increasing the homogenization time (A) would significantly (*p* < 0.05) reduce the PS, possibly due to longer homogenization shear force efficiently breaks the lipid globules into smaller particles. Similarly, increasing of solid lipid concentration (B) would lower the surface tension of particle, and reduce the PS. This could be attributed to Compritol 888 ATO^®^ (solid lipid) that comprises mono- (12–18% *w*/*w*), di- (45–54% *w*/*w*) and triglycerides (28–32% *w*/*w*) of fatty acid (C22 behenic acid) chains have surface active properties which would reduce the surface tension, and therefore producing smaller NLCs [12,13]. Likewise, the liquid lipid (C) also showed a significant negative impact (<0.05) on the PS, which could be associated with the different viscosity of liquid and solid lipid. Thus, increasing the liquid lipid concentration would drop the viscosity of NLCs, and reduce the surface tension, hence producing smaller NLCs [14]. In contrast, surfactant concentration (D) had a positive effect on PS, increasing its concentration would form larger NLCs. This could be due to the extra surfactant accumulated on the outer surface of NLCs. The nonpolar alkyl chain loop and tail of the surfactant formed a bridge in-between the nanoparticles, which might lead to aggregation [15,16,17]. The 3D response surface plot (Figure 1) shows the positive impact of AB, AC, BC interactions and the negative impact of AD, BD and CD interactions on PS of NLCs. The positive impact of AB interaction (increasing homogenization time and solid lipid concentration) or AC interaction (increasing homogenization time and liquid lipid concentration) or BC interaction (increasing solid lipid and liquid lipid concentrations) would increase the PS of NLCs, and form larger PS of NLCs. While the negative impact of AD interaction (increasing the homogenization time and surfactant concentration) or BD interaction (increasing solid lipid and surfactant concentrations) or CD interaction (increasing liquid-lipid and surfactant concentrations) would decrease the PS of NLCs, thus produced smaller PS of NLCs.

#### 3.1.2. Effect of Variables on the Polydispersity Index (PdI)

**Figure 2:**
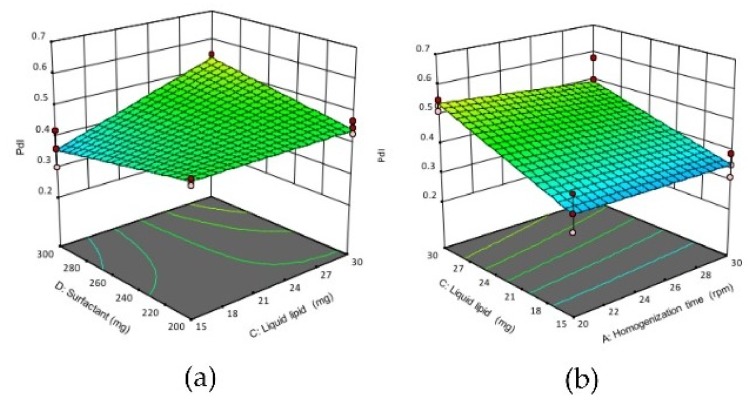

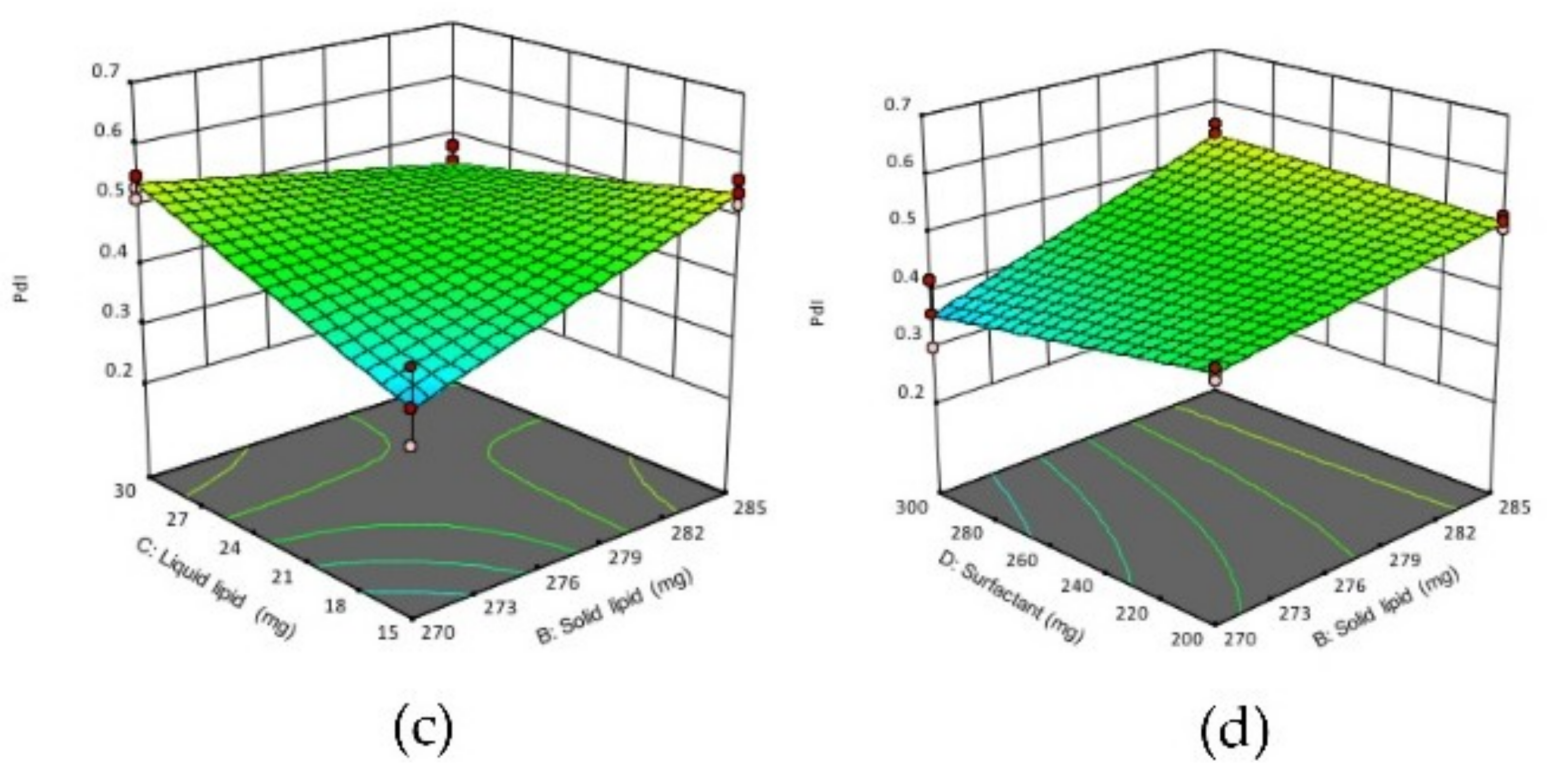
3D response surface plots showing the effect of interactions between (**a**) CD, (**b**) AC (**c**) BC and (**d**) BD on the PdI of NLCs. (**A**) homogenization time, (**B**) solid lipid concentration, (**C**) liquid lipid concentration, (**D**) surfactant concentration.

The PdI values of all formulations were ranging from 0.35 ± 0.04 to 0.54 ± 0.02 (Table 2). Equation (5) revealed factors B (solid lipid) and C (liquid lipid) have a positive impact on the PdI of NLCs. Accordingly, increasing the solid lipid concentration (B) or liquid lipid concentration (C) would increase the PdI significantly (*p* < 0.05). This could be due to the amount of surfactant was not sufficient to reduce the surface tension caused by increasing the lipid concentration, hence formed less homogeneous nanoparticles. Figure 2 shows the 3D response surface plot of the positive effect of AC, BC, BD and CD interactions on PdI of NLCs. The positive effect of AC interaction (increasing homogenization time and liquid lipid concentration) or BC interaction (increasing solid lipid and liquid lipid concentrations) or BD interaction (increasing solid lipid and surfactant concentrations), or CD interaction (increasing liquid lipid and surfactant concentration) would increase the PdI of NLCs, hence formed less homogenous nanoparticles.

#### 3.1.3. Effect of Variables on the Zeta Potential (ZP)

**Figure 3:**
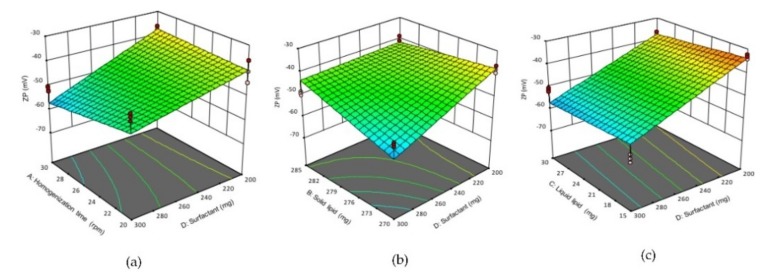
3D response surface plots showing the effect of interactions (**a**) AD, (**b**) BD (**c**), DC on ZP of NLCs. (**A**) homogenization time, (**B**) solid lipid concentration, (**C**) liquid lipid concentration, (**D**) surfactant concentration.

The ZP values of formulations were in the range from −33.2 ± 0.91 to −60.93 ± 1.64 mV (Table 2). The NLCs have the negative charge of ZP due to the ionization of glyceryl behenate moiety (a fatty acid in Compritol 888 ATO^®^). The homogenization time (A), solid lipid concentration (B) and surfactant concentration (C) showed a significant effect (*p* < 0.05) on ZP. According to Equation (6), factor A (homogenization time) showed a negative impact on ZP. Thus, prolonging homogenization time would increase ZP value. This could be due to longer homogenization shear force would enhance the ionization of glyceryl behenate. Similarly, factor D (surfactant concentration) also showed a negative impact on ZP. Increasing the surfactant concentration would enhance the emulsification of solid lipid, which led to higher ionization of glyceryl behenate moieties, hence increased ZP value. In contrast, factor B (solid lipid) has the positive impact on ZP, thus increasing the amount of solid lipid would reduce the ZP value, possibly due to incomplete emulsification of solid lipid by the available surfactant. The negative impact of factors AD and DC interactions and the positive impact of BD interactions on the ZP of NLCs are shown in the 3D surface plot (Figure 3). The negative impact of AD interaction (increasing homogenization time and surfactant concentration) or DC interaction (increasing surfactant and liquid lipid concentrations) would result in increasing the negative charge of the particles, hence increased the ZP value of NLCs. The positive effect of BD interaction (increasing solid lipid and surfactant concentration) would decrease the negative charge of nanoparticles thus decreased the ZP value of NLCs.

#### 3.1.4. Selection of NLC using the Desirability Function

The desirability function was used to select NLCs listed in Table 2. The following criteria, i.e., PS ≤ 150 nm, PdI ≤ 0.54 and ZP > −30 mV, was applied in the selection. The results showed that the NLC-1, NLC-4, NLC-7, NLC-8, NLC-12 and NLC-14 formulations had the highest desirability values of 0.824, 0.815, 0.816, 0.809, 0.815 and 0.752, respectively. The mean PS, PdI, and ZP of these formulations were in the range of 83.63 ± 6.55 nm to 119.8 ± 4.97 nm, 0.42 ± 0.01 to 0.50 ± 0.08 and −35.9 ± 5.68 mV to −51.23 ± 0.86 mV, respectively. Therefore, these formulations were selected for further study.

### 3.2. Determination of Encapsulation Efficiency and Yield

The selected NLC-1, NLC-4, NLC-7, NLC-8, NLC-12 and NLC-14 formulations were further studied by loading of 20 mg of LPV into NLCs (Table 3). It was found that the LPV-loaded NLCs formulation (LPV-NLC-4, LPV-NLC-7, and LPV-NLC-8) had the % EE > 92% and % yield > 96%. Thus, these formulations were further studied by incorporating of 30 and 35 mg of LPV. It was observed that increasing the concentration of LPV to 35 mg had decreased the % EE significantly (*p* < 0.05). Therefore, 30 mg of LPV was considered the highest amount could be loaded in the NLCs. Based on the % EE and % yield results, LPV-NLC-4, LPV-NLC-7, and LPV-NLC-8 formulations were selected for further study.

### 3.3. Freeze-Drying

Freeze-drying was conducted with the aim to improve the stability of LPV-NLCs by producing solid formulation. Cryoprotectant was used to protect the LPV-NLCs during freeze-drying. Therefore, several cryoprotectants were screened to evaluate their influence on PS and PdI of LPV-NLCs (Table 4). Among the screened cryoprotectant, trehalose was found superior in preventing LPV-NLCs aggregation during the freeze-drying process. Trehalose is cryoprotectant of choice for most of the biomolecules because it has some advantages compared to other types of sugars, such as lower hygroscopicity, less chemical interaction and lack of internal H-bonds which permits more flexible H-bond formation with nanoparticles during freeze-drying. Additionally, trehalose was reported to have a higher glass transition temperature which may help to prolong the nanoparticles stability and also effective cryoprotectant for compritol 888 ATO^®^ (glyceryl behenate) lipid-based nanoparticles during freeze-drying [18,19].

Freeze-drying of LPV-NLC-4, LPV-NLC-7 and LPV-NLC-8 formulations without trehalose produced a dried sticky sample with poor flowability, and difficult to redisperse in water. PS and PdI of the freeze-dried formulations were increased significantly (*p* < 0.05), but there was no change in %EE compared to the formulations before freeze drying (Table 5). In contrast, incorporation of different concentrations of trehalose after homogenization process had produced free-flowing formulations after tapping the bottle. The PS, PdI, and %EE of LPV-NLC-4-Tres, LPV-NLC-7-Tres and LPV-NLC-8-Tres significantly reduced (*p* < 0.05) in comparison to the freeze-dried formulations without trehalose. However, the addition of trehalose during homogenization had improved the %EE of formulations significantly (*p* < 0.05) compared to the addition of trehalose after homogenization. The LPV-NLC-7-Tres formulation had the lowest PS (286.8 ± 1.30nm) and the highest %EE (88.31 ± 2.04%) at the lipid: trehalose ratio of 1:4 (Table 6). The PS of LPV-NLC-7-Tres was significantly smaller (*p* < 0.05) than LPV-NLC-7. Increasing the size of LPV-NLC-7 could be due to the aggregation of nanoparticles during the freeze-drying process. The lower %EE of LPV-NLC-7-Tres compared to the LPV-NLC-7 could be due to the detachment of LPV from the outer surface of LPV-NLC-Tres formulations (Table 5). Therefore, lipid: trehalose 1:4 ratio was used in the preparation of freeze-dried LPV-NLC-4-Tres, LPV-NLC-7-Tres and LPV-NLC-8-Tres formulations for further investigation.

### 3.4. In Vitro Release Study

**Figure 4:**
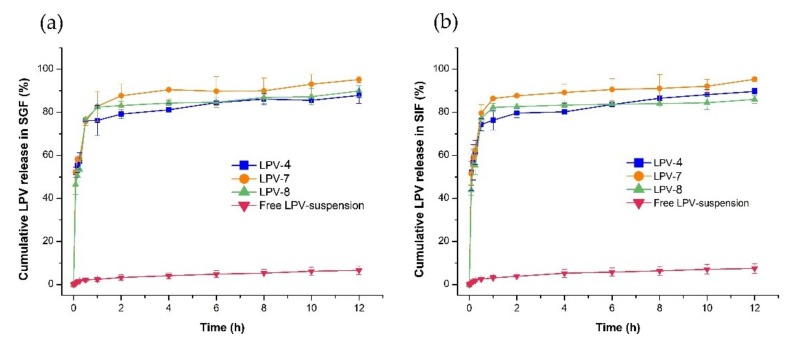
Comparison of LPV release from LPV-NLC-4-Tres, LPV-NLC-7-Tres, LPV-NLC-8-Tres, and LPV -suspension for 12 h in (**a**) SGF (pH 1.2) and (**b**) SIF (pH 6.8) media. Mean ± S.D, *n* = 3.

The release profile of LPV-NLC-4-Tres, LPV-NLC-7-Tres, and LPV-NLC-8-Tres in the SIF and SGF media showed more than 80% of LPV was released from the formulations (Figure 4a,b). The formulations showed a burst release pattern, and about 45% of LPV was released within 5 min and followed by a prolonged release in both release media. On the other hand, the LPV suspension showed 9.01 ± 1.41% and 9.65 ± 0.31% dissolution in the SIF and SGF media within 12 h, respectively.

The initial burst release of LPV from the LPV-NLC-Tres may be attributed to factors such as nanoparticles size provided a large surface area to the LPV-NLC-Tres formulation and also deposition of the attached LPV on the outer surface of the formulation. The latter might have occurred after the hot homogenization process whereby, the melted lipid first solidified and formed a central core of lipid and deposited some of the LPV on the outer surface of the lipid core. This, would provide a shorter diffusion distance for LPV released from the formulation surface into the media, which resulted in the burst release [20]. In addition, the presence of surfactants (poloxamer 188 and Tween^®^ 80) in the LPV-NLC-Tres formulation had facilitated the LPV release, while the nanosize range of particles in the formulation had contributed in enhancing the LPV solubility. In contrast, the LPV-suspension is hydrophobic in nature, thus it showed a limited solubility even in the presence of 1% poloxamer 188 in the release medium. Among the LPV-NLC-Tres formulations, the LPV-NLC-7-Tres showed the highest release (~97%) in both media and was selected as an optimized formulation for further study.

### 3.5. Differential Scanning Calorimetry (DSC)

**Figure 5:**
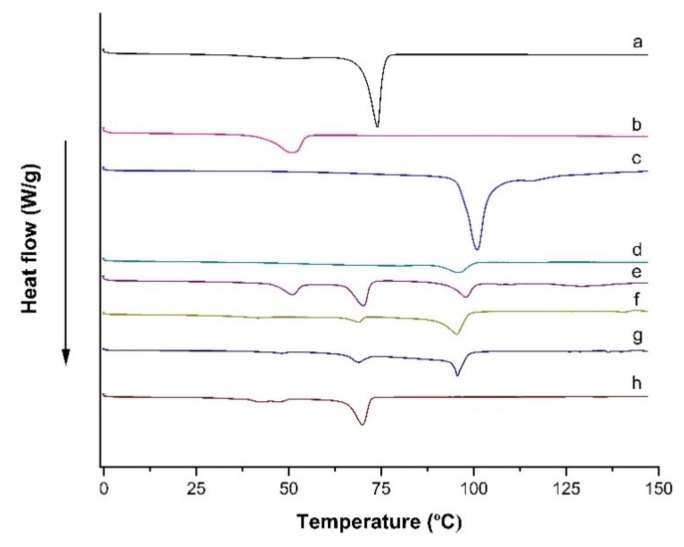
DSC thermograms scanned from 0 to 150 °C at a rate of 10 °C/min for (**a**) Compritol 888 ATO^®^, (**b**) Poloxamer 188, (**c**) Trehalose, (**d**) LPV, (**e**) Physical mixture (i.e., Compritol 888 ATO^®^, Poloxamer 188, trehalose and LPV), (**f**) Blank formulation, (**g**) LPV-NLC-7-Tres formulation, and (**h**) LPV-NLC-7 formulation.

DSC thermograms of bulk compritol 888 ATO^®^, poloxamer 188, LPV and trehalose exhibited melting peaks at 73.72 °C, 50.80 °C, 96.97 °C, and 100.43 °C, respectively (Figure 5). The thermogram of blank formulation, LPV-NLC-7, LPV-NLC-7-Tres showed that the Compritol 888 ATO^®^, poloxamer 188 and trehalose peaks in the formulation have shifted to 70.68 °C, 46.16 °C and 96.05 °C, respectively. The shifting of peaks to lower values might be attributed to a reduction in particle size associated with an increase in surface area and resulted in a decrease of enthalpy [15]. The thermogram of LPV-NLC-7 formulation was performed to find out whether the endothermic peak at 96.05 °C belongs to LPV and/or trehalose. The thermogram showed that there was no endothermic peak appeared at 96.05°C. Thus, this finding confirmed that the endothermic peak at 96.05 °C position in the thermogram of LPV-NLC-7-Tres formulation belongs to trehalose. The LPV endothermic peak was not noted in the thermogram of LPV-NLC-Tres formulation and LPV-NLC-7, which might be due to the LPV solubility had increased within the lipid matrix [1].

### 3.6. Wide Angle X-ray Scattering

**Figure 6:**
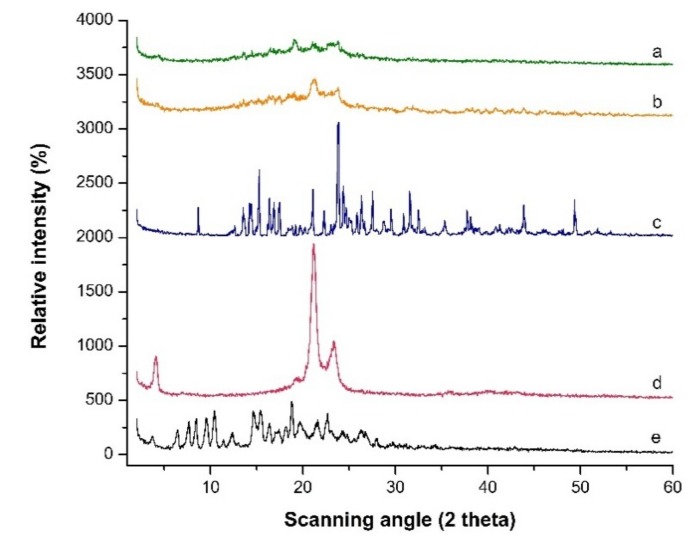
PXRD patterns scanned from 2° to 60° 2θ at a rate of 5 °C/min for (**a**) Freeze-dried LPV-NLC-7-Tres formulation, (**b**) Blank formulation, (**c**) Trehalose, (**d**) Compritol 888 ATO^®^ and (**e**) LPV.

The WAXS θ/2θ scans of LPV, compritol 888 ATO^®^, trehalose, blank formulation and LPV-NLC-7-Tres are shown in Figure 6. The LPV spectrum showed quite sharp and definite diffraction peaks at 10.4°, 15.4°, 18.8° and 22.6°, anticipated a crystalline nature [3]. However, these diffraction peaks have diminished in LPV-NLC-7-Tres formulation. This could be due to a reduction in the LPV crystallinity or increasing its amorphous state, suggested the enhancement of its solubility in lipids. Compritol 888 ATO^®^ showed sharp peaks at 21.1° and 23.3° angles, but the peaks were reduced in the LPV-NLC-7-Tres formulation due to polymorphic crystalline transformations of lipid (compritol 888 ATO^®^) upon heating. Aji Alex et al., reported a similar finding of LPV loaded in compritol 888 ATO^®^ based SLNs, whereby LPV decreased its crystallinity when loaded in SLNs. This suggested that the solubility of LPV had increased in the glyceryl behenate [1]. The crystalline peaks of trehalose at 8.72°, 15.27°, 21.07°, 23.87°, 26.32°, 27.52°, and 31.62° were reduced in the LPV-NLC-7-Tres formulation as compared to pure trehalose because it had formed an amorphous matrix with water molecules around the nanoparticles during freezing [21].

### 3.7. Evaluation using the Electron Microscope

**Figure 7:**
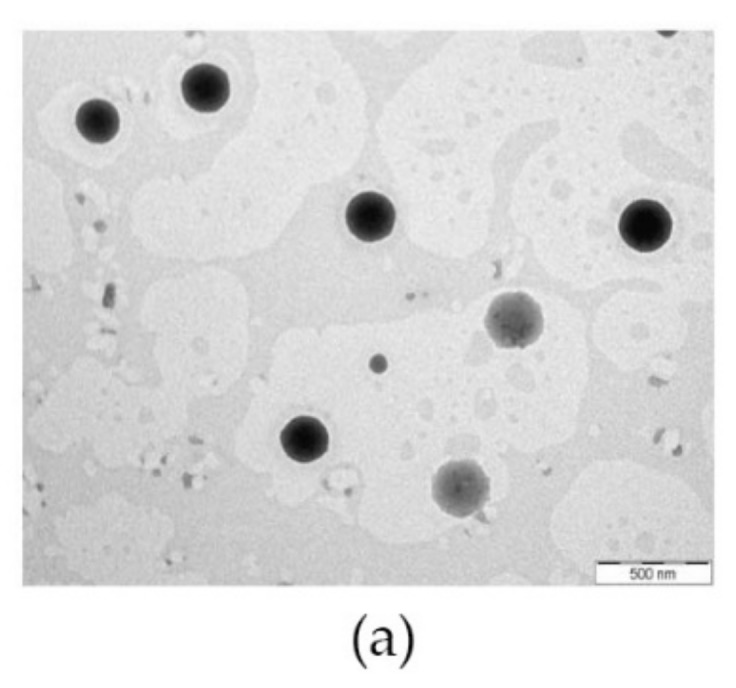

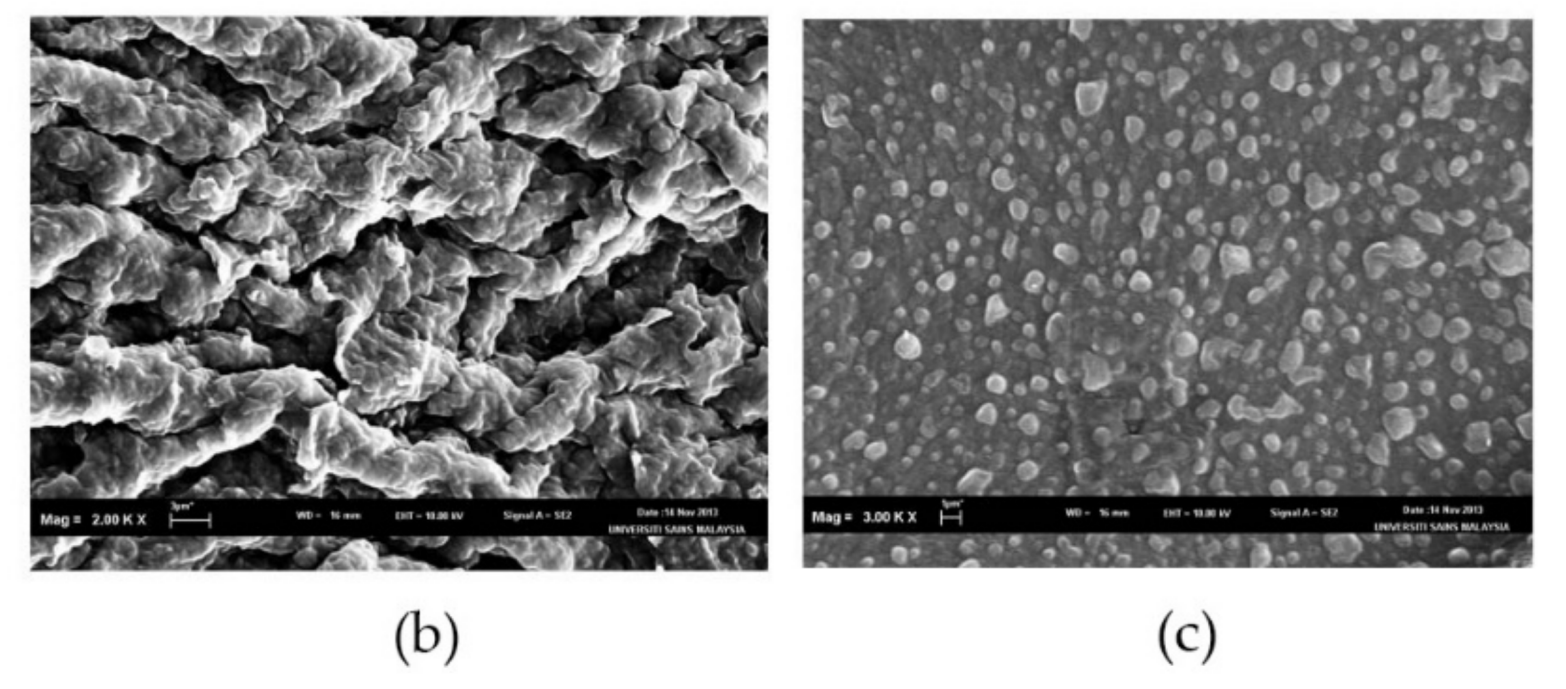
(**a**) TEM image of LPV-NLC-7 before freeze-drying; (**b**) SEM image of LPV-NLC-7 after freeze drying, and (**c**) SEM image of LPV-NLC-7-Tres after freeze drying.

The morphology of LPV-NLC-7 formulation before freeze-drying and after freez- drying was examined using transmission electron microscopy (TEM) and scanning electron microscopy (SEM), respectively. The nanoparticle of LPV-NLC before freeze-drying observed under TEM showed a spherical shape with the size of ~100nm. In contrast, the nanoparticles of freeze-dried LPV-NLC-7 (without trehalose) examined under SEM revealed that the particles had diffused and loss of their structure (Figure 7). However, freeze-drying of the formulation in the presence of trehalose (LPV-NLC-7-Tres) showed that the structure of particle was still preserved and the size of nanoparticle was ~200 nm.

### 3.8. Cellular Uptake of Optimized LPV-NLC-7-Tres Formulation

The monolayer model of the Caco-2 cell line is a popular model used for the investigation of drug cellular uptake across the intestinal epithelium cells [22]. Some researchers reported the in vitro cytotoxicity of commonly used excipients in pharmaceutical formulations namely Compritol 888 ATO [23], oleic acid [24,25], Tween^®^ 80 [26,27] and poloxamer 188 [28]. These excipients were also used in the present study. The maximum concentration of excipients, the cell line type and cell density used in the present study were within the safety range of their study. The maximum concentrations of Compritol 888 ATO^®^, oleic acid, Tween^®^ 80 and poloxamer 188 used in the present study were 285 mg, 30 mg, 150 mg, and 150 mg respectively. The reported oral LD50 of Compritol 888 ATO^®^, oleic acid, Tween^®^ 80 and poloxamer 188 were 5000 mg/kg, 25,000 mg/kg, 25,000 mg/kg and 42,200 mg/kg, respectively [29,30]. Thus, based on the above reports the concentrations of excipient used in the present study were also safe for the oral administration.

**Figure 8:**
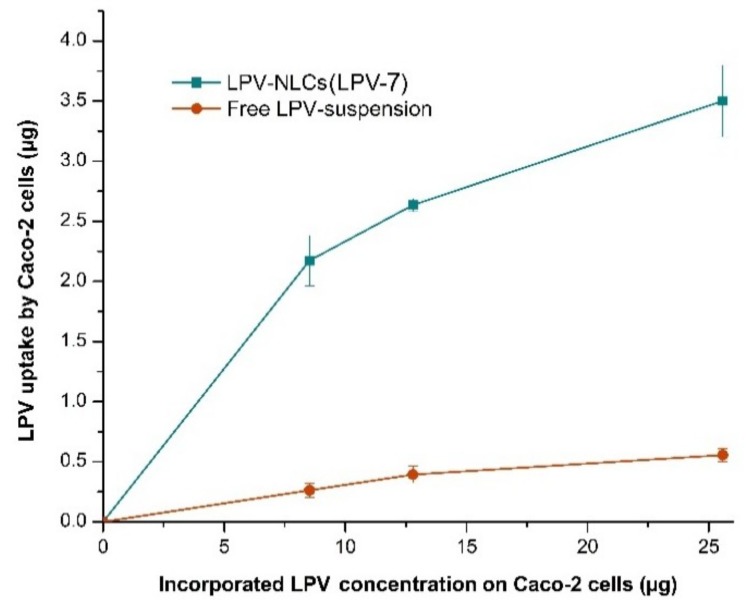
Comparison of LPV cellular uptake from LPV-NLC-7-Tres and LPV-suspension in Caco-2 cells. Mean ± SD, *n* = 3.

The present study showed that the Caco-2 cellular uptake was dependent on the concentration of LPV, whereby increasing the LPV concentration would increase its cellular uptake. Figure 8 shows the LPV cellular uptake from LPV-NLC-7-Tres and LPV suspension enhanced significantly (*p* < 0.05) from 2.17 µg/mL to 3.50 µg/mL and from 0.267 µg/mL to 0.555 µg/mL, respectively when LPV concentration was increased from 8.52 µg/mL to 25.58 µg/mL. The LPV cellular uptake from LPV-NLC-7-Tres was 6.30-fold higher than the LPV suspension. Higher cellular uptake of LPV-NLC-7-Tres could be due to increasing LPV solubility in the NLC formulation.

### 3.9. Stability Studies

The physical appearance of freeze-dried LPV-NLC-7-Tres samples stored at 5 ± 3 °C (refrigerated condition) was preserved, including flowability and ease of redispersion up to a six-month study period. Table 7 shows the PdI and ZP were not significantly changed (*p* > 0.05), but the PS was significantly increased (~40 nm), while the drug content was significantly reduced (~1.5%). In contrast, the samples stored at 25 ± 2 °C/60 ± 5% RH and 40 ± 2 °C/75 ± 5% RH conditions were found in aggregated compact solid form. When the samples were reconstituted in distilled water, the particles were difficult to re-disperse and aggregation was clearly seen visually. The PS and PdI were not measurable (NM) and the drug content decreased significantly (*p* < 0.05).

### 3.10. Oral Bioavailability Study

**Figure 9:**
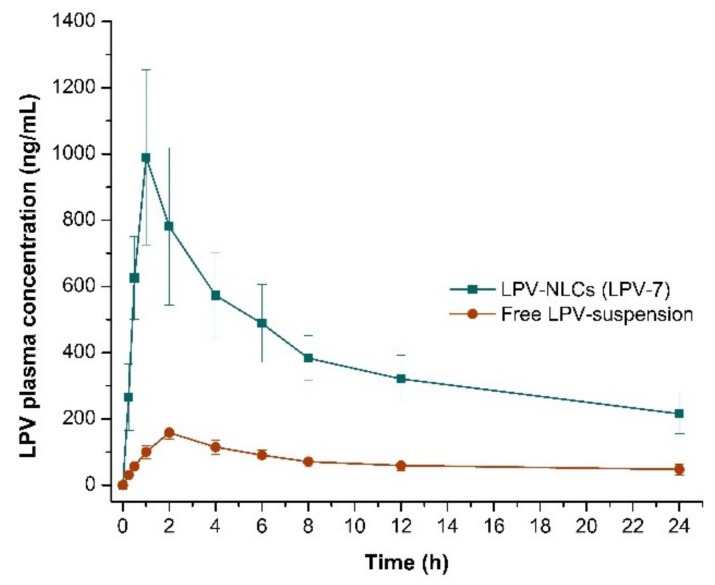
Comparison of LPV mean plasma concentration for 24 h following oral administration of LPV-NLC-7-Tres and LPV-suspension in rats. Mean ± S.D, *n* = 6.

The mean plasma concentration-time plot of LPV-NLC-7-Tres and LPV suspension are depicted in Figure 9. The concentration of LPV-NLC-7-Tres was higher than the LPV suspension in the plasma following oral administration. The *C*_max_ value of LPV-NLC-7-Tres (990.1 ± 264.7 ng/mL), was significantly higher (*p* < 0.05) than the LPV suspension (158.3 ± 18.5 ng/mL). The AUC_0-∞_ value of LPV-NLC-7-Tres (14635.1 ± 3847.5) was significantly (*p* < 0.05) higher than the LPV suspension (2094.5 ± 73.8). Thus, based on the AUC_0–∞_ value obtained, the bioavailability of LPV-NLC-7-Tres was 6.98-fold higher than the LPV suspension. The higher *C*_max_ and AUC_0-∞_ of LPV-NLC-7-Tres formulation could be due to an increase of LPV solubilization in the lipid matrix of the NLCs system. The result is in agreement with the finding in the cellular uptake experiment. In addition, the LPV-NLC-7-Tres has possibly bypassed the hepatic metabolism, and entered the lymphatic system. The lower *C*_max_ value of LPV-suspension might be due to LPV has low solubility, undergone extensive hepatic metabolism, and high pg-efflux. The LPV-NLC-7-Tres contains lipids (Compritol 888 ATO^®^ and oleic acid), thus, it might have passed the primary routes of lipid transport across the intestinal walls e.g., via transcellular absorption, paracellular transport, and stimulating chylomicrons secretion. The *T*_max_ of LPV-NLC-7-Tres (1 h) was significantly (*p* < 0.05) shorter than LPV suspension (2 h), which might be due to higher absorption of LPV via NLCs. The *t*_1/2_ of LPV-NLC-7-Tres (16.5 ± 2.36 h) was significantly (*p* < 0.05) longer than the LPV-suspension (5.4 ± 0.904 h). The elimination rate (*K*_e_) of LPV-NLC-7-Tres (0.0421 ± 0.007/h) was significantly (*p* < 0.05) slower than the LPV-suspension (0.121 ± 0.021/h), which indicated longer elimination of LPV from LPV-NLC-7-Tres formulation. Therefore, based on the pharmacokinetics results (Table 8), the present study had shown that NLCs enhanced the oral bioavailability of protease inhibitor drug, LPV.

## 4. Conclusions

The freeze-dried formulation of lopinavir-loaded nanostructured lipid carriers (LPV-NLC-7-Tres) was successfully developed and optimized using a 2^4^ full factorial design. The formulation had high cellular uptake and oral bioavailability. The enhancement of the oral bioavailability of lopinavir could be due to an increase in its solubility in the lipid matrix, and possibly bypass the hepatic metabolism and absorbed through the lymphatic system. The formulation was relatively stable under the refrigerated condition for six months.

## Figures and Tables

**Table 1:** Description of 2^4^ full factorial design.

**Factors (Independent Variables)**	**Low Level**	**High Level**
A: Homogenization time	20.00 min	30.00 min
B: Solid lipid (compritol 888 ATO^®^)	270.00 mg	285.00 mg
C: Liquid lipid (oleic acid)	15.00 mg	30.00 mg
D: Surfactants (poloxamer 188: Tween^®^ 80, 1:1)	200.00 mg	300.00 mg
**Responses (Dependent Variables)**	**Constraints**	
PS (nm)	Minimize	
PdI	Minimize	
ZP (mV)	Maximize	

**Table 2:** Optimization of independent variables and the responses of dependent variables.

Formula Code	Independent Variables				Dependent Variables Responses		
	(A) Homogenization Time (min)	(B) Solid lipid (mg)	(C) Liquid lipid (mg)	(D) Surfactant (mg)	PS (nm)	PdI	ZP (mV)
NLC-1	20	270	15	200	90.47 ± 2.55	0.44 ± 0.01	−36.4 ± 0.65
NLC-2	30	270	15	200	77.68 ± 2.29	0.44 ± 0.02	−33.2 ± 0.91
NLC-3	20	285	15	200	116.96 ± 4.09	0.52 ± 0.01	−42.9 ± 1.66
NLC-4	30	285	15	200	87.20 ± 1.79	0.42 ± 0.01	−41.6 ± 2.92
NLC-5	20	270	30	200	105.27 ± 11.29	0.45 ± 0.02	−40.3 ± 4.36
NLC-6	30	270	30	200	102.07 ± 32.26	0.39 ± 0.08	−38.0 ± 1.50
NLC-7	20	285	30	200	109.96 ± 5.81	0.44 ± 0.02	−48.4 ± 1.19
NLC-8	30	285	30	200	83.63 ± 6.55	0.47 ± 0.04	−41.0 ± 2.35
NLC-9	20	270	15	300	309.86 ± 56.24	0.36 ± 0.06	−54.1 ± 4.36
NLC-10	30	270	15	300	182.7 ± 62.66	0.35 ± 0.04	−60.9 ± 1.64
NLC-11	20	285	15	300	108.2 ± 7.90	0.54 ± 0.02	−35.1 ± 1.01
NLC-12	30	285	15	300	86.37 ± 4.49	0.44 ± 0.02	−35.9 ± 5.68
NLC-13	20	270	30	300	135.86 ± 8.15	0.53 ± 0.02	−43.8 ± 1.46
NLC-14	30	270	30	300	119.8 ± 4.97	0.50 ± 0.08	−51.2 ± 0.86
NLC-15	20	285	30	300	166 ± 7.37	0.45 ± 0.03	−48.9 ± 4.37
NLC-16	30	285	30	300	157.63 ± 32.16	0.46 ± 0.17	−49.5 ± 0.94

**Table 3:** Yield and entrapment efficiency of selected LPV-loaded NLCs formulations.

Formula Code	Drug Added (mg)	% Yield	% Encapsulation Efficiency
LPV-NLC-1	20	97.58 ± 0.56	70.7 ± 2.15
LPV-NLC-4	20	97.16 ± 0.98	97 ± 1.25
LPV-NLC-7	20	98.44 ± 1.23	92.6 ± 3.20
LPV-NLC-8	20	96.27 ± 1.11	96.75 ± 4.11
LPV-NLC-12	20	99.41 ± 0.68	81.1 ± 2.27
LPV-NLC-14	20	98.75 ± 0.75	83.35 ± 1.98
LPV-NLC-4	30	98.24 ± 0.44	95.66 ± 1.15
LPV-NLC-7	30	96.46 ± 0.27	90.53 ± 0.70
LPV-NLC-8	30	97.20 ± 0.30	92.23 ± 2.70
LPV-NLC-4	35	97.22 ± 0.18	80.47 ± 1.64
LPV-NLC-7	35	97.06 ± 0.28	80.35 ± 0.97
LPV-NLC-8	35	96.62 ± 0.18	79.60 ± 2.07

**Table 4:** Screening of cryoprotectants.

Cryoprotectants	Ratio (Lipid: Cryoprotectant)	Before Lyophilization		After Lyophilization	
		PS (nm)	PdI	PS (nm)	PdI
Mannitol	1:2	99.5 ± 0.900	0.309 ± 0.047	NM	1
	1:4			1799.9 ± 483.9	0.924 ± 0.132
	1:6			1901 ± 305.1	1
	1:8			1544.2 ± 205.2	1
Sorbitol	1:2			NM	1
	1:4			NM	1
	1:6			NM	1
	1:8			NM	1
Sucrose	1:2			837.0 ± 186.1	0.537 ± 0.411
	1:4			897.9 ± 72.5	0.913 ± 0.151
	1:6			965.2 ± 196.7	1
	1:8			1028.0 ± 125.9	1
Trehalose	1:2			744.2 ± 210.1	0.460 ± 0.300
	1:4			383.7 ± 19.2	0.586 ± 0.370
	1:6			463.1 ± 4.7	0.512 ± 0.215
	1:8			478.4 ± 13.8	0.591 ± 0.030

Abbreviations: NM, not measurable.

**Table 5:** Influence of cryoprotectant addition after homogenization process on PS, PdI, ZP and %EE.

Formula Code	Ratio (Lipid: Trehalose)	PS (nm)	PdI	ZP (mV)	% EE
**Before Freeze Drying**					
LPV-NLC-4	1:0	93.6 ± 0.4	0.307 ± 0.016	−43.6 ± 1.45	96.87 ± 0.76
LPV-NLC-7	1:0	104.3 ± 0.6	0.383 ± 0.012	−48.2 ± 1.50	91.72 ± 0.68
LPV-NLC-8	1:0	92.5 ± 0.6	0.315 ± 0.030	−42.5 ± 1.50	93.72 ± 0.96
**After Freeze Drying**					
LPV-NLC-4	1:0	NM	1	−42.5 ± 0.53	95.66 ± 1.15
LPV-NLC-4-Tres	1:1	606.4 ± 98.9	0.361 ± 0.428	−42.7 ± 0.36	71.91 ± 3.03
	1:2	472.3 ± 698.8	0.758 ± 0.210	−43.3 ± 1.49	68.99 ± 1.86
	1:4	399.4 ± 1.5	0.720 ± 0.062	−42.3 ± 1.38	68.67 ± 0.60
	1:6	487.5 ± 8.6	0.233 ± 0.112	−41.6 ± 1.45	82.61 ± 1.80
LPV-NLC-7	1:0	NM	0.603 ± 0.447	−47.1 ± 1.00	90.53 ± 0.70
LPV-NLC-7-Tres	1:1	1381.1 ± 383.2	0.898 ± 0.176	−45.4 ± 0.95	69.80 ± 1.87
	1:2	859.8 ± 330.4	0.649 ± 0.501	−46.6 ± 1.25	68.13 ± 1.4
	1:4	402.9 ± 11.7	0.277 ± 0.052	−48.2 ± 1.75	71.49 ± 2.14
	1:6	445.0 ± 51.4	0.531 ± 0.152	−46.3 ± 1.80	78.76 ± 1.60
LPV-NLC-8	1:0	NM	1	−41.4 ± 1.27	92.23 ± 2.70
LPV-NLC-8-Tres	1:1	472.0 ± 2.3	0.342 ± 0.199	−41.3 ± 1.21	66.77 ± 3.70
	1:2	398.6 ± 2.4	0.295 ± 0.143	−42.7 ± 0.47	64.25 ± 1.95
	1:4	386.5 ± 9.7	0.296 ± 0.026	−42.5 ± 0.80	61.05 ± 1.86
	1:6	425.4 ± 1.6	0.399 ± 0.059	−40.6 ± 0.83	66.44 ± 1.94

Abbreviations: NM, not measurable; LPV-NLC-4, LPV-NLC-7 and LPV-NLC-8 formulations without trehalose; LPV-NLC-4-Tres, LPV-NLC-7-Tres and LPV-NLC-8-Tres formulations containing trehalose.

**Table 6:** Influence of cryoprotectant addition during homogenization process on PS, PdI, ZP and %EE.

Formula Code	Ratio (Lipid: Trehalose)	After Freeze Drying			
		PS (nm)	PdI	ZP (mV)	% EE
LPV-NLC-4-Tres	1:1	935.5 ± 1.50	0.468 ± 0.032	−43.8 ± 2.96	79.12 ± 1.32
	1:2	750.2 ± 1.20	1	−41.5 ± 1.24	78.43 ± 3.21
	1:4	337.5 ± 8.00	0.475 ± 0.145	−41.7 ± 1.15	79.38 ± 5.93
	1:6	920.4 ± 2.50	0.798 ± 0.211	−42.6 ± 0.78	84.54 ± 1.17
LPV-NLC-7-Tres	1:1	NM	1	−48.2 ± 0.95	80.32 ± 1.33
	1:2	969.9 ± 1.90	1	−47.3 ± 1.00	79.54 ± 3.24
	1:4	286.8 ± 1.30	0.413 ± 0.017	−48.6 ± 0.89	88.31 ± 2.04
	1:6	775.5 ± 160.11	0.957 ± 1.21	-46.6 ± 0.50	89.15 ± 4.33
LPV-NLC-8-Tres	1:1	1244.6 ± 536.36	1	-42.2 ± 0.95	76.23 ± 2.67
	1:2	1082.52 ± 483.93	1	-41.6 ± 0.98	75.24 ± 3.28
	1:4	335.8 ± 3.10	0.525 ± 0.037	-40.6 ± 0.55	84.93 ± 1.62
	1:6	870.42 ± 343.70	1	-42.6 ± 1.45	77.43 ± 4.94

Abbreviations: NM, not measurable; LPV-NLC-4-Tres, LPV-NLC-7-Tres and LPV-NLC-8-Tres formulations containing trehalose.

**Table 7:** The stability of optimized LPV-NLCs-7-Tres formulation stored at 5 ± 3 °C, 25 ± 2 °C/60 ± 5% RH and 40 ± 2 °C/75 ± 5% RH (*n* = 3).

**Stability at 5 ± 3 °C**				
**Parameters**	**0 month**	**1 month**	**3 months**	**6 months**
PS (nm)	255.8 ± 1.4	275.5 ± 5.7	286.5 ± 11.2	292.5 ± 9.8
PdI	0.531 ± 0.085	0.589 ± 0.115	0.543 ± 0.112	0.552 ± 0.112
ZP (mV)	−48.50 ± 0.60	−48.30 ± 1.51	−47.10 ± 1.20	−47.50 ± 2.75
Drug content (%EE)	100.06 ± 0.07	99.93 ± 0.66	98.91 ± 0.61	97.82 ± 0.62
**Stability at 25 ± 2 °C/60 ± 5% RH**				
**Parameters**	**0 month**	**1 month**	**3 months**	**6 months**
PS (nm)	255.8 ± 1.4	800.6 ± 159.31	NM	NM
PdI	0.531 ± 0.085	1	NM	NM
ZP (mV)	−48.50 ± 0.60	−45.50 ± 1.63	−47.50 ± 1.42	−42.10 ± 2.66
Drug content (%EE)	100.06 ± 0.069	92.89 ± 1.008	88.93 ± 0.372	84.61 ± 0.715
**Stability at 40 ± 2 °C/75 ± 5% RH**				
**Parameters**	**0 month**	**1 month**	**3 months**	**6 months**
PS (nm)	255.8 ± 1.4	933.93 ± 312.01	NM	NM
PdI	0.531 ± 0.085	1	NM	NM
ZP (mV)	−48.50 ± 0.60	−46.90 ± 1.77	−45.60 ± 1.20	−45.70 ± 3.65
Drug content (%EE)	100.06 ± 0.07	76.64 ± 0.62	71.32 ± 5.53	68.54 ± 11.74

Abbreviations: NM, not measurable.

**Table 8:** Pharmacokinetic parameters of LPV-NLC-7-Tres and free LPV-suspension after oral administration.

Formulation	Pharmacokinetic Parameters				
	AUC_0-∞_ ± SD (ng-h/mL)	*C*_max_ ± SD (ng/mL)	*T*_max_ ± SD (h)	*t*_1/2_ ± SD (h)	*K*_e_ ± SD (h^−1^)
LPV-NLC-7-Tres	14635.1 ± 3847.5	990.1 ± 264.7	1 ± 0.00	16.5 ± 2.36	0.0421 ± 0.007
Free LPV-suspension	2094.5 ± 473.8	158.3 ± 18.5	2 ± 0.00	5.4 ± 0.904	0.121 ± 0.021

Abbreviations: AUC_0-∞_, area under the plasma concentration-time curve; *C*_max_, maximum plasma concentration; *T*_max,_ time taken to reach maximum plasma concentration; *t*_1/2_, elimination half-life; *K*e, elimination rate.
